# Histone H3K9 demethylase JMJD2B induces hepatic steatosis through upregulation of PPARγ2

**DOI:** 10.1038/s41598-018-31953-x

**Published:** 2018-09-13

**Authors:** Ji-Hyun Kim, Dae Young Jung, Arulkumar Nagappan, Myeong Ho Jung

**Affiliations:** Division of Longevity and Biofunctional Medicine & Healthy Aging Korean Medical Research Center, School of Korean Medicine, Pusan National University, 49 Busandaehak-ro, Mulgeum-eup, Yangsan-si, Gyeongnam 50612 Republic of Korea

## Abstract

Understanding the epigenetic mechanisms underlying the progression of hepatic steatosis is important for identifying new therapeutic targets against nonalcoholic fatty liver disease (NAFLD). We investigated the functional role of histone demethylase JMJD2B in the pathologic regulation of hepatic steatosis. JMJD2B expression was markedly increased in HepG2 cells treated with palmitate and oleate or liver X receptor agonist T09013178 and in the liver of high-fat diet (HFD)-induced obese mice. Overexpression of JMJD2B using adenovirus in HepG2 cells stimulated the expression of peroxisome proliferator-activated receptor γ2 (PPARγ2) and its steatosis target genes associated with fatty acid uptake and lipid droplet formation, resulting in increased intracellular triglyceride (TG) accumulation. Conversely, knocking down JMJD2B using siRNA reversed JMJD2B-mediated effects in HepG2 cells. The JMJD2B-dependent upregulation of PPARγ2 was associated with the removal of di- and trimethylation of histone H3 lysine 9 on the promoter of PPARγ2. Furthermore, exogeneous expression of JMJD2B using adenovirus in mice resulted in hepatic steatosis when fed a HFD, which was accompanied with increased expression of hepatic PPARγ2 and its steatosis target genes. Together, our results provide novel insights into the pivotal role of JMJD2B in the development of hepatic steatosis through upregulation of PPARγ2 and steatosis target genes.

## Introduction

Nonalcoholic fatty liver disease (NAFLD) is a common chronic hepatic disorder and an emerging health concern worldwide. NAFLD has been linked with an increasing incidence of metabolic diseases, which include insulin resistance, type 2 diabetes, and hypertriglyceridemia^1^. Hepatic steatosis, which features excessive accumulation of triglyceride (TG) accumulation, is a characteristic of NAFLD and occurs from increased synthesis (lipogenesis) and uptake of fatty acids in the liver, decreased β-oxidation of fatty acids and export of very low-density lipoprotein from the liver, and continuous lipolysis in adipocytes^2^. Hepatic steatosis progresses into steatohepatitis, inflammation, fibrosis, cirrhosis, and hepatocellular cancer^2^. Thus, understanding the pathological mechanisms underlying the progression of hepatic steatosis is important for identifying new therapeutic targets against NAFLD. Recently, the number of studies revealing the important role of epigenetics in the pathogenesis of hepatic steatosis has increased. However, epigenetic regulation of hepatic steatosis by histone methylation remains poorly understood.

Methylation of histone lysine residues regulates chromatin formation and functions as a crucial epigenetic mechanism to regulate gene expression^3^. Although methylation of histone H3 at lysine 4 (H3K4) and of histone H3 at lysine 36 (H3K36) positions is primarily associated with active transcription, methylation of histone H3 at lysine 9 (H3K9) and of histone H3 at lysine 27 (H3K27) positions and histone H4 at lysine 20 (H4K20) are associated with gene repression^4^. Specific histone methyltransferases or demethylases are responsible for methylation or demethylation, respectively, at each amino acid position^5^. Several histone demethylases have been identified and classified into two classes: FAD-dependent amine oxidases (LSD demethylases) and Fe(II)- and α-ketoglutarate-dependent Jumonji C (JmjC) domain-containing demethylase (JMJD demethylase)^6^. LSD demethylases containing LSD1 and LSD2 demethylate mono- and dimethylated H3K4 and H3K9. The substrate specificity for H3K4, H3K9, H3K27, or H3K36 has been used to classify JmjC domain-containing histone demethylases into many subfamilies^6^. The JMJD2 or KDM4 family consists of JMJD2A (KDM4A), JMJD2B (KDM4B), and JMJD2C (KDM4C). They demethylate di- and trimethylated H3K9 and H3K36 (H3K9me2/me3 and H3K36me2/me3)^7^. JMJD2B specifically catalyzes the removal of di- and trimethylated H3K9 (H3K9me2/me3), converting both histone marks to the monomethylated state. Thus, JMJD2B functions as a transcriptional activator^7^. Recent studies have shown that JMJD2B expression is largely upregulated in various cancers and correlates with larger tumor size and advanced clinical stage^8^. JMJD2B mediates neoplastic transformation of estrogen-positive cells and increases transcription of many hypoxia-inducible genes in cancer cell lines^9,10^. Therefore, JMJD2B likely plays an important role in facilitating tumorigenesis.

Peroxisome proliferator-activated receptorγ (PPARγ) is present in two isoforms: PPARγ1 and PPARγ2^11^. While PPARγ1 is expressed in many tissues, PPARγ2 is predominantly expressed in adipose tissue^12^. PPARγ2 is well known as a master adipogenic transcription factor that induces the formation of lipid droplets through stimulation of adipogenic genes in adipocytes^13^. PPARγ2 also plays a key role as a hepatic steatosis transcription factor^14^. The increased expression of PPARγ2 has been observed in livers of obese rodents^15,16^ and NAFLD patients^17^. Hepatic PPARγ2 stimulates the uptake of hepatic fatty acids, re-esterification of fatty acids into monoacylglycerol, and formation of lipid droplets through upregulation of fatty acid translocase (CD36), fatty acid-binding protein 4 (FABP4), monoacylglycerol acyltransferase 1 (MOGAT1), perilipin 2 (PLIN2), and fat-specific protein 27/cell death-inducing DFFA-like effector C (FSP27/CIDEC), which promote hepatic steatosis. Epigenetic regulation of PPARγ2 has been recently reported to be associated with hepatic steatosis^18,19^. The histone H3K4 methyltransferase MLL4 enhances the expression of PPARγ2 and its hepatosteatosis target genes and results in the induction of hepatic steatosis^18^. In addition, histone deacetylase3 (HDAC3) binds to retinoic acid receptor-related orphan receptor α (RORα) on the promoter of PPARγ2 and represses PPARγ2 expression in hepatocytes, thereby protecting against diet-induced hepatic steatosis^19^. We previously reported that JMJD2B promotes PPARγ2 expression via erasing H3K9me2/me3 on the PPARγ2 promoter, which stimulates adipogenesis in 3T3-L1 preadipocytes^20^; however, the role of JMJD2B in the development of hepatic steatosis has not been elucidated.

Therefore, in this study, we investigated the functional role of JMJD2B in the pathogenesis of hepatic steatosis *in vitro* and *in vivo*. To investigate this, intracellular TG accumulation and expression of PPARγ2 and its steatosis target genes were measured in JMJD2B-overexpressing or -knockdown HepG2 cells. Enrichment of H3K9me2 and H3K9me3 on PPARγ2 was examined in JMJD2B-overexpressing HepG2 cells. Furthermore, induction of hepatic steatosis *in vivo* was assessed in JMJD2B-overexpressing mice.

## Results

### JMJD2B expression is increased in hepatosteatosis cell and animal models

**Figure 1:**
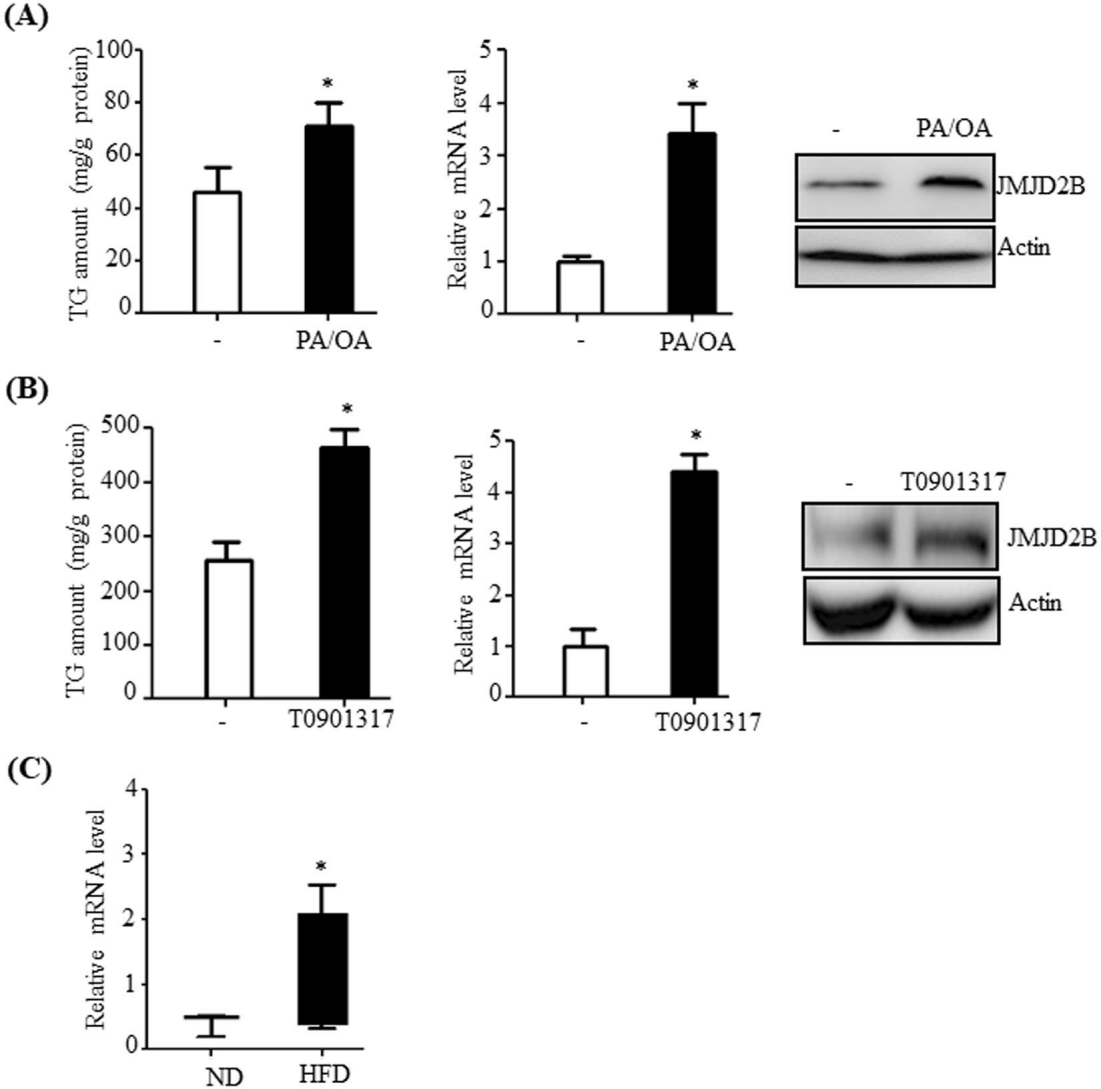
JMJD2B expression increases in hepatic steatotic cell and animal models. (**A**) HepG2 cells were incubated with a mixture of palmitic acid (PA) and oleic acid (OA) (1:2 ratio) at 800 μM concentrations for 24 h, and intracellular triglyceride (TG) levels were analyzed by a TG assay kit. JMJD2B mRNA and protein levels were examined by qPCR and western blotting, respectively. Data represent means ± SEM of three independent experiments performed in triplicate. *p < 0.05 vs. no treatment. The full-length western blots corresponding to truncated blots are presented in Supplementary Figure S1A. (**B**) HepG2 cells were treated with T0901317 (10 μM) for 24 h, and intracellular triglyceride (TG) levels were measured by a TG assay kit. JMJD2B mRNA and protein levels were examined by qPCR and western blotting, respectively. Data represent means ± SEM of three independent experiments performed in triplicate. *p < 0.05 vs. no treatment. The full-length western blots are presented in Supplementary Figure S1B. (**C**) Total RNAs were isolated from the livers of HFD-induced obese mice. The JMJD2B mRNA levels were assessed by qPCR. Data represent means ± SEM of 5 mice. *p < 0.05 vs. ND mice. ND: normal diet. HFD: high fat diet.

To determine whether JMJD2B expression is correlated with NAFLD pathogenesis, we examined JMJD2B expression in hepatosteatosis cell and animal models. To produce a hepatosteatosis cell model, HepG2 cells were treated with a mixture of palmitic acid (PA) and oleic acid (OA) (molar ratio of 1:2). Treatment with PA/OA increased intracellular TG levels in HepG2 cells compared with those in non-treated HepG2 cells (Fig. 1A). Concomitant with increased TG levels, the expressions of JMJD2B were increased at the mRNA and protein levels in PA/OA-treated HepG2 cells (Fig. 1A). LXR signaling contributes to the development of hepatic steatosis^21^. Administration of LXR agonist T0901317 to mice induces severe fatty liver^21^. Hence, we examined JMJD2B expression under the pathogenic conditions of hepatic steatosis induced by LXR signaling. Treatment with T0901317 also induced JMJD2B expression in HepG2 cells, which is consistent with increased TG level (Fig. 1B). Moreover, we evaluated the JMJD2B expression in the livers of HFD-induced obese mice. qPCR revealed that JMJD2B mRNA levels were higher in the livers of HFD-induced obese mice than in the livers of lean mice (Fig. 1C). These results suggest that JMJD2B expression is correlated with hepatic steatosis.

### Overexpression of JMJD2B increases intracellular TG levels and stimulates PPARγ2 expression in HepG2 cells

**Figure 2:**
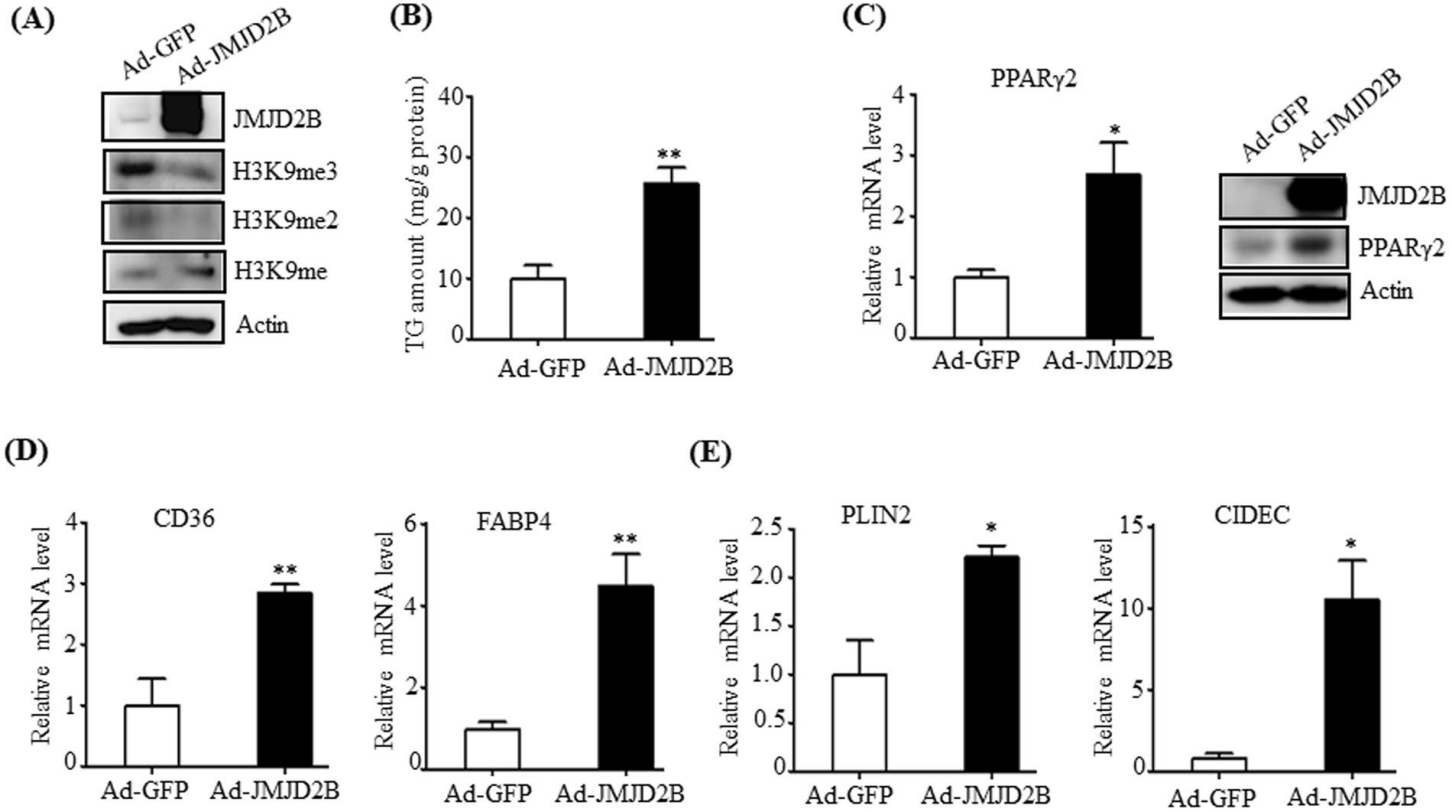
Overexpression of JMJD2B increases intracellular TG levels and stimulates PPARγ2 expression in HepG2 cells. HepG2 cells in 6-well plates were infected with Ad-GFP or Ad-JMJD2B. (**A**) The levels of JMJD2B, H3K9me, H3K9me2, and H3K9me3 were measured by western blotting. The full-length western blots corresponding to truncated blots are provided in Supplementary Figure S2A. (**B**) Intracellular TG levels were measured by TG assay kit. (**C**) PPARγ2 expression was assessed by qPCR and western blotting. The full-length western blots corresponding to truncated blots are presented in Supplementary Figure S2B. (**D**,**E**) The expression of PPARγ2 steatosis target genes was evaluated by qPCR. Data represent means ± SEM of three independent experiments performed in triplicate. *p < 0.05, **p < 0.01 vs. Ad-GFP.

We then investigated the functional role of JMJD2B in hepatic steatosis. To test this, a gain-of-function study was performed in HepG2 cells. To overexpress JMJD2B ectopically in HepG2 cells, HepG2 cells were infected with adenovirus containing JMJD2B (Ad-JMJD2B) and intracellular TG levels were determined. To verify JMJD2B overexpression in Ad-JMJD2B-infected HepG2 cells, we measured the levels of JMJD2B and its target histone marks in cell extracts. Western blotting revealed that JMJD2B protein levels were significantly increased in Ad-JMJD2B-infected HepG2 cells compared with those in Ad-GFP-infected HepG2 cells (Fig. 2A). Consistent with the increased JMJD2B protein level, Ad-mediated JMJD2B overexpression led to decreased H3K9me2 and H3K9me3 global epigenetic marks but increased H3K9me (Fig. 2A), demonstrating that JMJD2B was successfully overexpressed in Ad-JMJD2B-infected HepG2 cells. We then measured intracellular TG levels in Ad-JMJD2B-infected HepG2 cells. Quantitation of TG levels revealed that overexpressing JMJD2B increased intracellular TG levels in HepG2 cells (Fig. 2B), suggesting that JMJD2B contributes to the induction of hepatic steatosis. We then tried to find a target gene of JMJD2B that would induce hepatic steatosis. PPARγ2 is well known as a hepatosteatosis transcription factor as well as an adipogenic transcription factor^9,10^. Accordingly, we examined the expression of PPARγ2 as a JMJD2B target gene in Ad-JMJD2B-infected HepG2 cells. As shown in Fig. 2C, overexpressing JMJD2B increased PPARγ2 mRNA levels. Western blotting also revealed that PPARγ2 protein level was significantly enhanced by JMJD2B overexpression (Fig. 2C). Then, we measured the expression of steatosis target genes of PPARγ2. Concomitant with the increase in PPARγ2 expression, overexpressing JMJD2B stimulated the expression of hepatosteatosis genes including fatty acid uptake-associated genes *CD36*, *FABP4* (Fig. 2D) and lipid droplet-associated genes *PLIN2, CIDEC* (Fig. 2E) in HepG2 cells, which are known PPARγ2 steatosis target genes. Collectively, the results indicate that JMJD2B stimulates the expression of PPARγ2 and its steatosis genes, which may contribute to the development of hepatic steatosis.

### Knockdown of JMJD2B reduces the intracellular TG level and represses PPARγ2 expression in HepG2 cells

**Figure 3:**
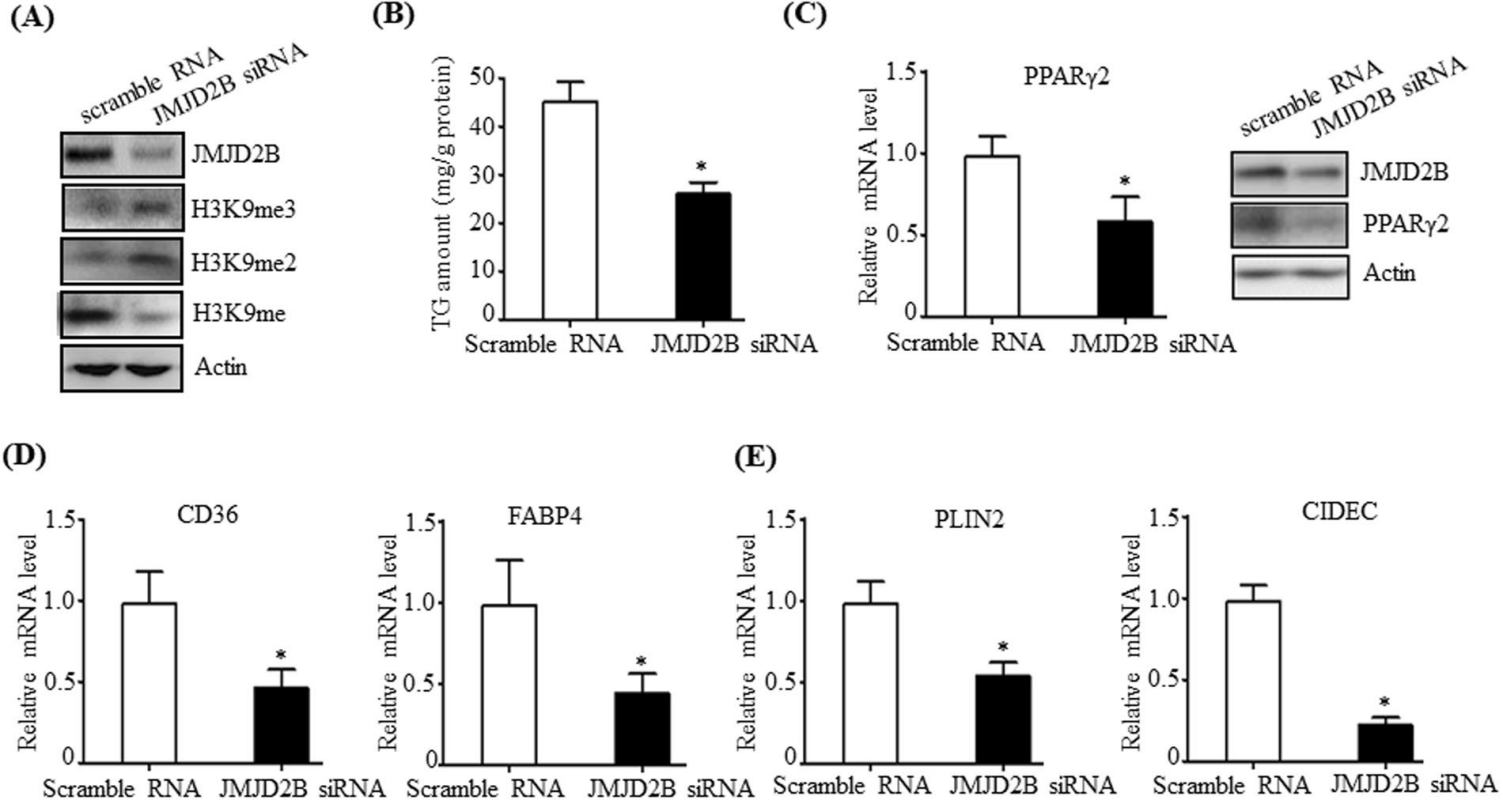
Knockdown of JMJD2B reduces the intracellular TG level and represses PPARγ2 expression in HepG2 cells. HepG2 cells in 6-well plates were transfected with scramble RNA or JMJD2B siRNA. (**A**) The levels of JMJD2B, H3K9me, H3K9me2, and H3K9me3 were measured by western blotting. The full-length western blots corresponding to truncated blots are presented in Supplementary Figure S3A. (**B**) Intracellular TG levels were measured by a TG assay kit. (**C**) PPARγ2 expression was assessed by qPCR and western blotting. The full-length western blots corresponding to truncated blots are presented in Supplementary Figure S3B. (**D**,**E**) The expression of PPARγ2 steatosis target genes was evaluated by qPCR. Data represent means ± SEM of three independent experiments performed in triplicate. *p < 0.05 vs. scramble RNA.

To further confirm the functional role of JMJD2B in hepatic steatosis, a loss-of-function experiment was performed in HepG2 cells. To knock down JMJD2B in HepG2 cells, HepG2 cells were transfected with JMJD2B siRNA, and intracellular TG levels were determined. To confirm the knockdown of JMJD2B, we measured the levels of JMJD2B and its target histone markers in JMJD2B siRNA-transfected HepG2 cells. Western blotting revealed that JMJD2B siRNA efficiently reduced JMJD2B protein level (Fig. 3A). Consistent with the reduced JMJD2B protein level, JMJD2B knockdown increased the H3K9me2 and H3K9me3 global epigenetic marks (Fig. 3A), demonstrating that JMJD2B was successively knocked down in JMJD2B siRNA-transfected HepG2 cells. We then measured intracellular TG levels in JMJD2B siRNA-transfected HepG2 cells. As shown in Fig. 3B, JMJD2B knockdown significantly reduced intracellular TG levels in HepG2 cells. Then, we assessed the expression of PPARγ2 and its steatosis target genes in JMJD2B siRNA-transfected HepG2 cells. As shown in Fig. 3C, JMJD2B knockdown resulted in a decrease in mRNA and protein levels of PPARγ2. Concomitant with reduced PPARγ2 expression, the expression of its steatosis target genes including *CD36*, *FABP4* (Fig. 3D) *and PLIN2, CIDEC* (Fig. 3E) was also reduced.

### JMJD2B reduces the enrichment of H3K9me2 and H3K9me3 on the promoter of PPARγ2

**Figure 4:**
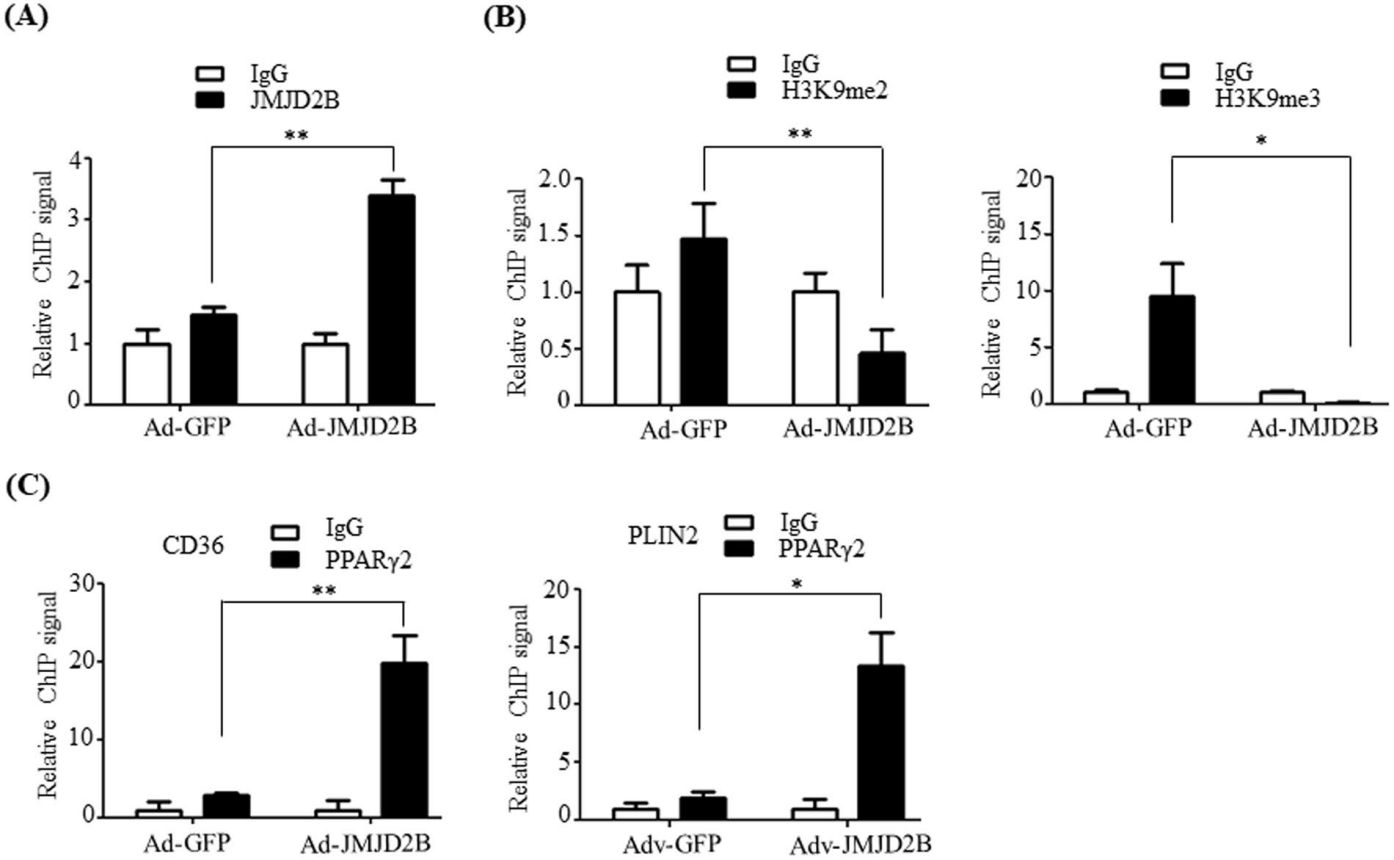
JMJD2B lessens the enrichment of H3K9me2 and H3K9me3 on PPARγ2 promoters and increases the binding of PPARγ2 to PPREs on promoters of *CD36* and *PLIN2*. HepG2 cells were infected with adenovirus Ad-GFP or Ad-JMJD2B. (**A**) The recruitment of JMJD2B to the PPARγ2 promoter was analyzed by ChIP-qPCR. (**B**) The enrichment of H3K9me2 and H3K9me3 on promoters of PPARγ2 was analyzed by ChIP-qPCR. (**C**) The recruitment of PPARγ2 to PPRE on promoters of *CD36* and *PLIN2* was assessed by ChIP-qPCR. Data represent means ± SEM of three independent experiments performed in triplicate. *p < 0.05, **p < 0.01 vs. Ad-GFP.

Next, we explored the possible mechanism by which JMJD2B upregulates PPARγ2 expression. Since JMJD2B is a histone H3K9me2/me3 demethylase, we hypothesized that JMJD2B demethylates both H3K9me2 and H3K9me3 on the promoter of PPARγ2 and stimulates PPARγ2 expression. Thus, we first examined the recruitment of JMJD2B to the promoter of PPARγ2 and the enrichment of H3K9me2 and H3K9me3 on the promoter of PPARγ2 in JMJD2B-overexpressing HepG2 cells. As shown in Fig. 4, ChIP-qPCR revealed that overexpressing JMJD2B resulted in a significant increase in JMJD2B recruitment to the PPARγ2 promoter compared with that in the control Ad-GFP-infected HepG2 cells (Fig. 4A), whereas the enrichment of H3K9me2 and H3K9me3 on the promoter of PPARγ2 was reduced in Ad-JMJD2B-infected HepG2 cells (Fig. 4B), indicating that JMJD2B removes the repressive histone marks H3K9me2 and H3K9me3 on the promoter of PPARγ2, which thereby stimulates PPARγ2 expression.

We then investigated whether JMJD2B-mediated upregulation of PPARγ2 subsequently increases the binding of PPARγ2 to PPARγ response elements (PPREs) on the promoters of PPARγ2 steatosis target genes to stimulate their expression. The binding of PPARγ2 to PPREs on the promoter of PPARγ2 steatosis target genes was measured in Ad-JMJD2B-infected HepG2 cells by ChIP-qPCR. ChIP-qPCR revealed that PPARγ2 binding on PPREs of *CD36* and *PLIN2* promoters increased significantly in Ad-JMJD2B-infected HepG2 cells compared with that in Ad-GFP-infected HepG2 cells (Fig. 4C). Taken together, these results indicate that JMJD2B stimulates PPARγ2 expression via removal of H3K9me and H3K9me3 on the PPARγ2 promoter, which subsequently stimulates the expression of PPARγ2 steatosis target genes by its increased binding to PPRE on their promoters.

### Adenovirus-mediated JMJD2B overexpression induces hepatic steatosis *in vivo*

**Figure 5:**
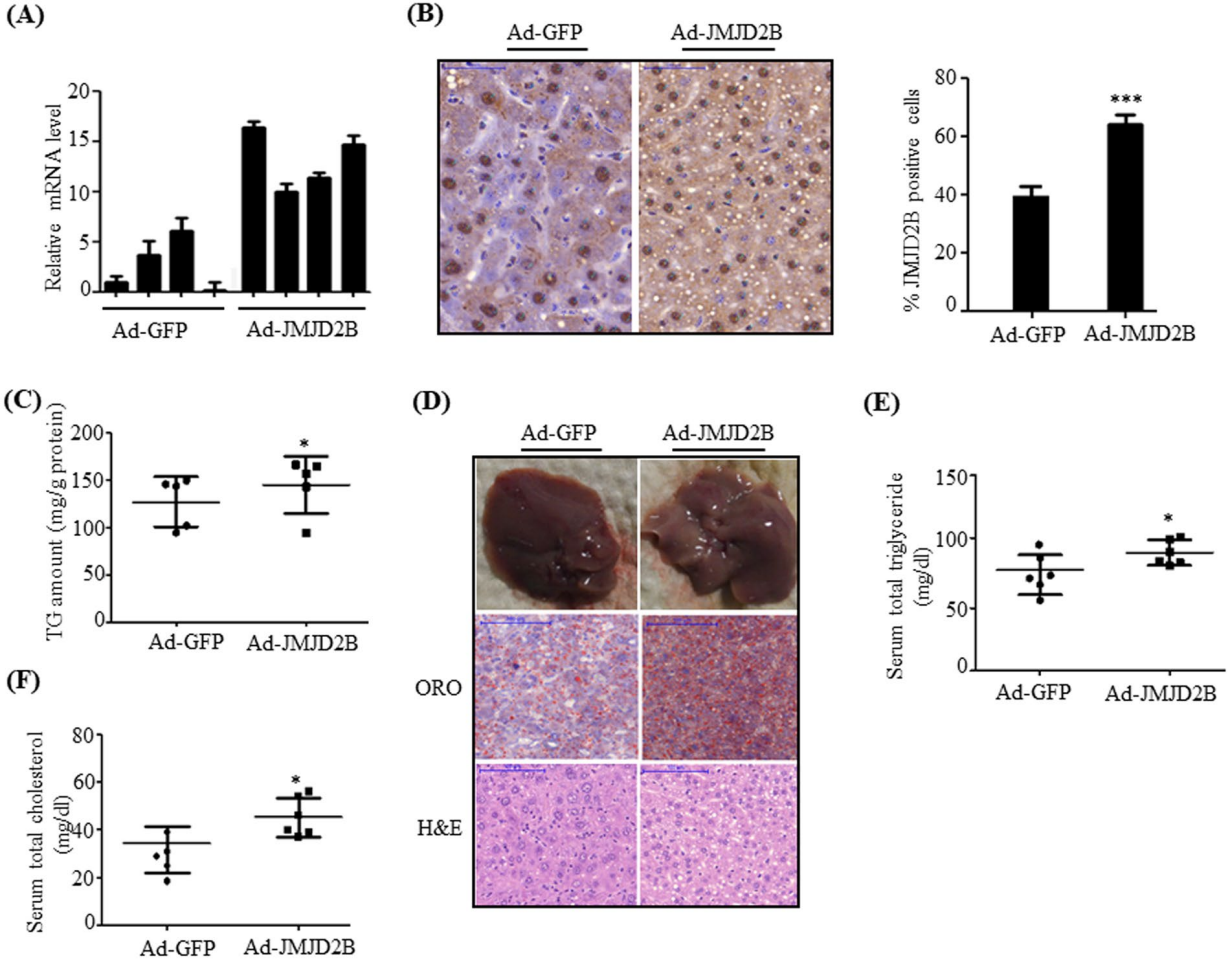
Adenovirus-mediated JMJD2B overexpression stimulated hepatic steatosis *in vivo*. C57BL/6 mice (8 weeks old) were injected with adenovirus Ad-GFP or Ad-JMJD2B. After injection, Ad-injected mice were fed a HFD for 2 weeks. (**A**) JMJD2B expression was determined by qPCR (**B**) Immunostaining of JMJD2B (scale bar = 50 μm). Representative photographs are shown. JMJD2B positive cells were counted in ten random areas at 400× magnification and analyzed by using image J. Quantification of immunostaining assay is represented as percentage of JMJD2B positive cells. (**C**) Hepatic triglyceride (TG) levels were measured by a TG assay kit. (**D**) Liver morphology, ORO and H&E staining (scale bar = 100 μm). Representative images are presented. (**E**) Serum total TG levels. (**F**) Serum total cholesterol levels. Data represent means ± SEM from 5 mice. *p < 0.05 vs. Ad-GFP-infected mice.

To further substantiate the functional role of JMJD2B in hepatic steatosis *in vivo*, we injected recombinant Ad-JMJD2B through the tail vein in C57BL/6J mice to overexpress hepatic JMJD2B. The mice were fed a HFD for 2 weeks after the injection. qPCR revealed that JMJD2B was successfully overexpressed in the livers of Ad-JMJD2B-injected mice (Fig. 5A). Immunostaining also confirmed increased JMJD2B overexpression (Fig. 5B). We then investigated the effect of JMJD2B on the development of hepatic steatosis in Ad-JMJD-injected mice. Quantitation of TG revealed that hepatic TG levels significantly increased in Ad-JMJD2B-injected mice compared with those in Ad-GFP-injected mice (Fig. 5C) which agrees with *in vitro* data that JMJD2B overexpression increased intracellular TG levels in HepG2 cells. Consistent with increased TG levels, Ad-JMJD2B injected mice showed a white-colored fatty liver compared with relatively healthy liver in Ad-GFP injected mice (Fig. 5D). ORO staining also confirmed that hepatic TG levels markedly increased in Ad-JMJD2B-injected mice compared with those in Ad-GFP-injected mice (Fig. 5D). Furthermore, H&E staining revealed that Ad-JMJD2B-injected mice showed more lipid droplets compared than Ad-GFP-injected mice (Fig. 5D). Consistent with the observed phenotypes, biochemical analysis revealed that serum levels of total TG (Fig. 5E) and cholesterol (Fig. 5F) increased in Ad-JMJD2B-injected mice compared with those in Ad-GFP-injected mice. Taken together, these results demonstrated that *in vivo* JMJD2B overexpression in the liver histologically and biologically stimulates hepatic steatosis in mice fed a HFD.

**Figure 6:**
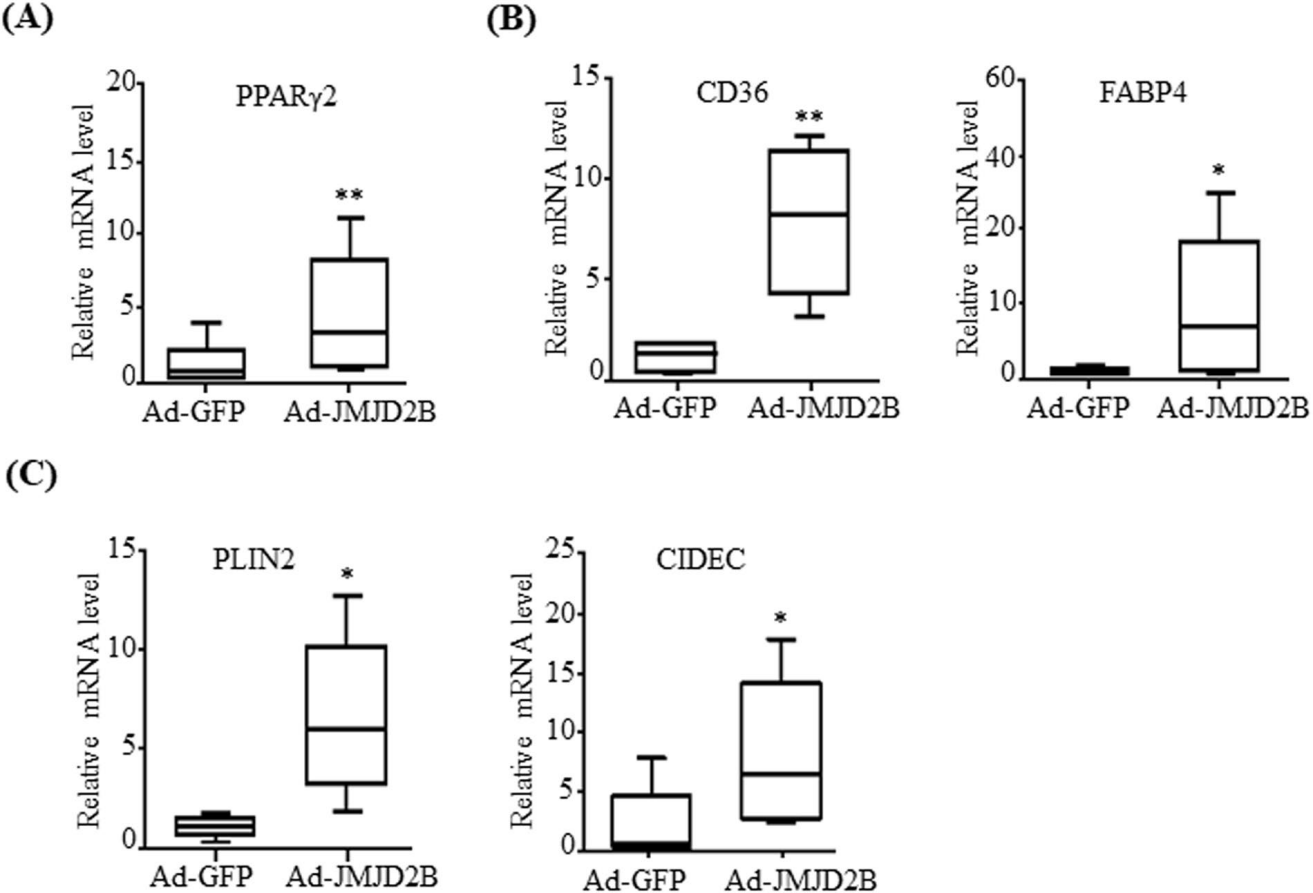
Adenovirus-mediated JMJD2B overexpression promotes the expression of PPARγ2 and its steatosis target genes in mouse liver. C57BL/6 mice (8 weeks old) were injected with adenovirus Ad-GFP or Ad-JMJD2B. After injection, Ad-injected mice were fed a HFD for 2 weeks. (**A**) PPARγ2 expression was measured by qPCR. (**B**,**C**) The expression of PPARγ2 steatosis target genes was measured by qPCR. Data represent means ± SEM from 5 mice. *p < 0.05, **p < 0.01 vs. Ad-GFP-infected mice.

Next, we examined the expression of hepatic PPARγ2 and its steatosis genes in the livers of Ad-JMJD2B-injected mice. Consistent with *in vitro* data, *in vivo* JMJD2B overexpression also increased mRNA levels of PPARγ2 (Fig. 6A) and its steatosis genes (Fig. 6B and C) in the livers of Ad-JMJD2B-injected mice.

### Gomisin N reduces the expression of JMJD2B and PPARγ2 in the livers of HFD-induced obese mice

**Figure 7:**
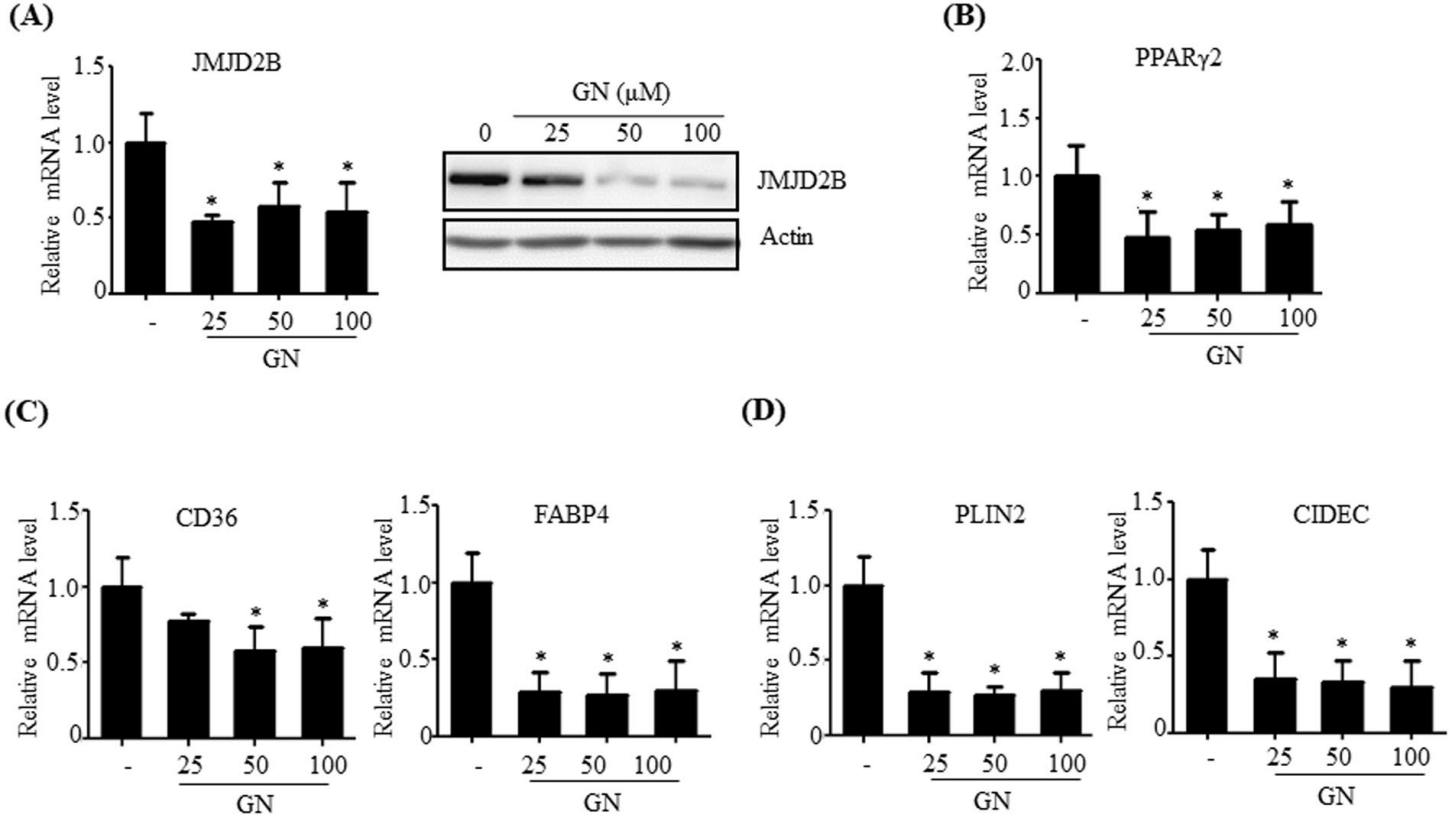
Gomisin N (GN) represses the expression of JMJD2B, PPARγ2, and PPARγ2 steatosis target genes in HepG2 cells. HepG2 cells were treated with GN (100 μM) for 12 h. (**A**) JMJD2B expression was assessed by qPCR and western blotting. The full-length western blots corresponding to truncated blots are given in Supplementary Figure S4. (**B**) PPARγ2 expression was measured by qPCR. (**C**,**D**) The expression of PPARγ2 steatosis target genes was assessed by qPCR. Data represent means ± SEM of three independent experiments performed in triplicate. *p < 0.05 vs. no treatment.

Gomisin N (GN) is a lignan isolated from *Schisandra chinensis*^22^. In our previous study, we demonstrated that GN alleviates HFD-induced hepatic steatosis via AMP-activated protein kinase (AMPK) activation^22^. In the present study, to further examine the possible mechanism of GN against HFD-induced hepatic steatosis, we investigated whether GN downregulates JMJD2B and subsequently represses the expression of PPARγ2 and its steatosis genes. HepG2 cells were treated with GN and the expression of JMJD2B, PPARγ2, and its steatosis genes was determined by qPCR. As shown in Fig. 7, GN treatment reduced mRNA and protein levels of JMJD2B (Fig. 7A). Furthermore, consistent with reduced JMJD2B expression, mRNA levels of PPARγ2 (Fig. 7B) and its steatotic target genes (Fig. 7C and D) were also reduced. These results suggest that GN-mediated downregulation of JMJD2B and PPARγ2 may play a role in ameliorating HFD-induced hepatic steatosis.

## Discussion

Epigenetic regulation by environmental factors, such as poor nutrition, physical inactivity, and weight gain, can contribute to the development and progression of several metabolic diseases, including hepatic steatosis. Therefore, elucidating the epigenetic mechanisms by which hepatic steatosis develops can provide a new perspective on the pathogenesis of NAFLD and a potential drug target for this disease. In the present study, we investigated the functional role of histone demethylase JMJD2B in pathologic mechanisms by which NAFLD is developed. In the present study, we found that JMJD2B promotes PPARγ2 expression by removing repressive H3K9me2 and H3K9me3 marks on the promoter of PPARγ2, which subsequently increases the expression of PPARγ2 steatosis target genes and results in the development of hepatic steatosis.

Histone modifications, including acetylation, methylation, phosphorylation, and ubiquitination, are essential in regulating chromatin structure and gene expression. Aberrant histone modifications prelude the development of various diseases, including cancer and insulin resistance. Recent studies demonstrated that HDAC3 negatively regulates hepatic steatosis by regulating lipid metabolism genes via several mechanisms^19,23,24^. HDAC3 represses the expression of lipogenesis genes by deacetylating their promoter regions and thereby prevents hepatic steatosis^23^. Recently, other mechanisms involved in HDAC3-mediated inhibition of hepatic steatosis have been elucidated^19,24^. HDAC3 specifically binds to ROR, which is recruited to the promoter of PPARγ2, and represses PPARγ2 expression^19^. In addition, HDAC3 also interacts with prospero-related homeobox 1 protein (PROX1), which is corecruited to genes associated with lipid homeostasis via hepatocyte nuclear factor 4α, and regulates the expression of genes for lipid synthesis and lipolysis^24^. Depleting HDAC3 or PROX1 in the liver increases hepatic TGs and results in hepatic steatosis. However, epigenetic regulation of hepatic steatosis by histone methylation is largely unknown. A recent study demonstrated that the histone H3K4 methyltransferase MLL4 is recruited to the PPREs of PPARγ2 and steatosis genes, which stimulates their expression and results in hepatic steatosis^18^. In the present study, we attempted to identify a new epigenetic regulation mechanism of hepatic steatosis by histone methylation.

Histone H3K9 demethylase JMJD2B is a member of the JMJD2 family. It specifically recognizes H3K9me2 and H3K9me2/3, converting H3K9me2/3 to a monomethylated status. Recently, we reported that JMJD2B promotes PPARγ2 expression and activates adipogenesis in 3T3-L1 preadipocytes^20^; however, the role of JMJD2B in hepatic steatosis remains unclear. Therefore, in the present study, we investigated the functional role of JMJD2B in hepatic steatosis. We performed gain-of-function and loss-of-function studies in HepG2 cells to see whether JMJD2B induces hepatic steatosis. We observed that Ad-mediated JMJD2B overexpression resulted in increased intracellular TG accumulation in HepG2 cells. In contrast, JMJD2B knockdown using siRNA resulted in significantly decreased intracellular TG levels in HepG2 cells, suggesting that JMJD2B plays a role in inducing hepatic steatosis. We then attempted to identify a hepatosteatosis target transcription factor of JMJD2B. As mentioned, PPARγ2 is now regarded as a hepatic steatosis transcription factor as well as an adipogenic transcription factor^13,14^. Liver-specific PPARγ2 stimulates the expression of genes associated with fatty acid uptake (*CD36*, *FABP4*), lipid droplet formation (*PLIN2*, *CIDEC*), and fatty acid esterification mediated by the monoacylglycerol pathway^14^. Our previous study revealed that JMJD2B upregulates PPARγ2 expression in 3T3-L1 adipocytes by erasing H3K9me2/3 on the promoter of PPARγ2^20^; therefore, we believed that PPARγ2 might be a hepatosteatosis target transcription factor of JMJD2B. In the present study, we observed that JMJD2B overexpression stimulated PPARγ2 expression in HepG2 cells, whereas JMJD2B knockdown repressed PPARγ2 expression. In addition, concomitant with increased PPARγ2 expression, JMJD2B overexpression promoted the expression of PPARγ2 steatosis target genes, including *CD36*, *FABP4*, *PLIN2* and *CIDEC*. Furthermore, the ChIP assay revealed that binding of PPARγ2 on PPREs of steatosis target genes, such as *CD36* and *PLIN2*, was enhanced in JMJD2B-overexpressing HepG2 cells. Together, these results indicate that JMJD2B promotes PPARγ2 expression, which subsequently increases the expression of PPARγ2 steatosis target genes and results in increased intracellular TG accumulation.

We further ascertained the functional role of JMJD2B in the pathogenesis of hepatic steatosis *in vivo*. We found that mice injected with recombinant Ad-JMJD2B showed elevated hepatic TG accumulation, which was revealed by quantifying TG levels and ORO and H&E staining. The results indicate the potential involvement of JMJD2B in the progression of hepatic steatosis. Furthermore, we confirmed JMJD2B–PPARγ2 signaling in the livers of Ad-JMJD2B-injected mice. Consistent with the results in HepG2 cells, the expression of PPARγ2 and its steatosis target genes *CD36*, *FABP4*, *PLIN2*, and *CIDEC2* was enhanced in the livers of Ad-JMJD2B-injected mice compared with those in the livers of Ad-GFP-injected mice; therefore, our *in vivo* data also showed that JMJD2B is an epigenetic regulator in the development of hepatic steatosis. The present observation of the significant elevation of JMJD2B expression in the livers of HFD-induced obese mice concomitant with increased PPARγ2 expression provided evidence of the crucial role of JMJD2B–PPARγ2 signaling in HFD-induced hepatic steatosis.

We further determined the mechanism by which JMJD2B promotes PPARγ2 expression. It has been reported that di-and trimethylation of H3K9 is associated with the repression of PPARγ2 in adipocyte cells^25^. To clarify the mechanism by which JMJD2B promotes PPARγ2 expression in HepG2 cells, we determined changes in H3K9me2 and H3K9me3 levels on the PPARγ2 promoter in JMJD2B-overexpressed HepG2 cells. ChIP-qPCR revealed that enrichment of H3K9me2 and H3K9me3 on the promoter of PPARγ2 was decreased in JMJD2B-overexpressed HepG2 cells in contrast to increased recruitment of JMJD2B, indicating that JMJD2B erases repressive histone markers H3K9me2 and H3K9me3 on the promoter of PPARγ2, resulting in the upregulation of PPARγ2.

Repressive H3K9me3 is located together with active H3K4me3 as an assumed bivalent locus on PPARγ2^26^. Sufficient H3K4 methylation for histone H3K4 methyltransferase is required for H3K9 demethylation^26^. As previously stated, MLL4-mediated trimethylation of H3K4 on the PPARγ2 promoter stimulates PPARγ2 and its steatotic target gene expression through H3K4me3^18^. Combined with these results, removal of H3K9me3 by JMJD2B, as shown in our present study, might play a key role in increasing the active marker H3K4me3 on the promoter of PPARγ2 in HepG2 cells to stimulate PPARγ2 expression; therefore, further studies should examine H3K4me3 in JMJD2B-overexpressing HepG2 cells.

A previous study demonstrated that JMJD2B interacts with C/EBPβ on the promoters of C/EBPβ-targeted genes and demethylates H3K9me3 on C/EBPβ target genes, resulting in the activation of C/EBPβ target gene expression in 3T3-L1 adipocytes^25^. This suggests that C/EBPβ recruits JMJD2B to the promoter of PPARγ2 to remove H3K9me2 and H3K9me3. We found that C/EBP consensus binding sites exist around the transcription initiation site on the promoter of PPARγ2; therefore, we assumed that C/EBPβ was a candidate transcription factor to recruit JMJD2B into the promoter region of PPARγ2. Accordingly, we plan to study and elucidate the interaction of JMJD2B with C/EBPβ in HepG2 cells in the future.

Epigenetic mechanisms involved in disease progression serve as therapeutic targets; therefore, identifying epigenetic modifiers will provide major advances in the treatment of NAFLD. The results of the present study suggest that downregulation of JMJD2B–PPARγ2 signaling might attenuate hepatic steatosis, so compounds that inhibit JMJD2B–PPARγ2 signaling can be notable therapeutic resources against this disease. We previously reported that GN ameliorates HFD-induced hepatic steatosis^22^. In the present study, we determined whether GN inhibits JMJD2B–PPARγ2 signaling, which might contribute to the attenuation of hepatic steatosis. In HepG2 cells, GN efficiently decreased JMJD2B expression and subsequently reduced the expression of PPARγ2 and its steatotic target genes, which suggests the possible contribution of the GN-mediated inhibition of JMJD2B–PPARγ2 signaling to the progression of HFD-induced hepatic steatosis. Thus, preventing JMJD2B–PPARγ2 signaling may represent a potential therapeutic strategy against NAFLD.

In conclusion, JMJD2B plays a pivotal role in the development of hepatic steatosis by upregulating PPARγ2 and its steatosis target genes. Overall, the findings of the present provide novel insights into the pivotal role of epigenetic regulation of hepatic steatosis and a new candidate therapeutic target against hepatic steatosis.

## Methods

### Reagents

Dulbecco’s modified Eagle medium (DMEM), penicillin–streptomycin, and fetal bovine serum (FBS) were obtained from HyClone Laboratories Inc. (Logan, UT, USA). The antibody against JMJD2B (NB100-74605) was purchased from Novus Biologicals (Littelton, CO, USA). Antibodies against H3K9me (07–450), H3K9me2 (07–441), and H3K9me3 (07–442) were obtained from Millipore (Billerica, MA, USA). Antibodies against PPARγ2 (sc-166731), and β-actin (sc-47778) were purchased from Santa Cruz Biotechnology (Santa Cruz, CA, USA). Sodium palmitate (P09767), bovine serum albumin (A6003) and oleic acid (O3008) were purchased from Sigma-Aldrich (St. Louis, MO, USA).

### Cell culture

HepG2 human hepatocellular carcinoma cell line was purchased from the American Type Culture Collection (Manassas, VA, USA). The cells were maintained in DMEM supplemented with 10% heat-inactivated FBS, penicillin (20 U/mL), and streptomycin (20 μg/mL). HepG2 cells were treated with a mixture of PA and OA (1:2) or T0901317 (10 μM) for 24 h. PA and OA were dissolved overnight in 10% fatty acids-free bovine serum albumin (BSA) and in serum-free DMEM at a stock concentration of 800 mM, respectively. PA and OA (1:2 molar ratio) were added to the culture medium at final concentration of 800 μM.

### Infection of HepG2 cells with recombinant adenovirus

Adenovirus vectors (Ad-GFP and Ad-JMJD2B) encoding green fluorescent protein (GFP) and JMJD2B were purchased by Vector Biolabs (Malvern, PA, USA). Ad-GFP or Ad-JMJD2B was added at a multiplicity of infection (MOI) of 100 to infect HepG2 cells. The infected HepG2 cells were washed and replaced with fresh media. At 48 h post-infection, the cells were rinsed, lysed, and used for further experiments.

### Transfection of HepG2 cells with siRNAs

The siRNA targeting JMJD2B was obtained from Integrated DNA Technologies (Coralville, Iowa, USA) (sense: 5′-CCAGUUCAGUAUCAAUUAAAGCCCG-3′, antisense: 5′-CGGGCUUUAAUUGAUACUGAACUGGAG-3′). Interferin transfection reagent (Polyplus-transfection Inc., New York, NY, USA) was used to transfect the HepG2 cells with siRNAs.

### Total RNA preparation and quantitative real-time polymerase chain reaction (qPCR)

Total RNA from HepG2 cells and mice samples was isolated using TRIZOL (Invitrogen, Carlsbad, CA, USA) according to the manufacturer’s recommendations. One microgram total RNA was used for cDNA synthesis using the GoScript Reverse Transcription System (Promega, Madison, WI, USA). qPCR was carried using a SYBR Green premixed Taq reaction mixture with gene-specific primers. The primer sequences are provided in Supplementary Table S1.

### Western blotting

The protein lysates (20 μg) from HepG2 cell or liver tissues were subjected to 10% SDS-polyacrylamide gel electrophoresis (SDS-PAGE) and the resolved proteins were transferred to polyvinylidene difluoride membranes (Millipore, Billerica, MA, USA). The membranes were incubated with primary antibodies (1:1000 dilution) for JMJD2B, H3K9me, H3K9me2, H3K9me3, PPARγ2 and β-actin at 4 °C overnight followed by incubation with horseradish peroxidase-conjugated secondary antibody (1:1000 dilution) for 1 h at room temperature. Protein bands were visualized using an enhanced chemiluminescence (ECL) western blot detection kit (Amersham, Uppsala, Sweden).

### Chromatin immunoprecipitation (ChIP)-qPCR

ChIP was performed as previously described^13^. Briefly, HepG2 cells were chemically crosslinked in 1% formaldehyde for 10 min at room temperature. The crosslinked chromatin was sonicated to shear it into 400-bp fragments using a Bioruptor sonicator (Diagenode, Denville, NJ, USA). For immunoprecipitation, samples were incubated with 1–2 μg of antibodies against JMJD2B, H3K9me2, H3K9me3, and PPARγ2, or non-specific IgG control in the presence of secondary antibody conjugated to Dynabeads (Invitrogen, Carlsbad, CA, USA). After purification of DNA, qPCR was performed using the following primers: PPARγ2 sense 5′**-** GTACAGTTCACGCCCTCAC**-**3′, PPARγ2 antisense 5′**-** TGGCAAGACTTGGTACATTACA **-**3′; CD 36 sense 5′- GTGTGCCTTTTGCATCTTGA-3′, CD 36 antisense 5′- GGGGCACTAACAGAAAACGA-3′; PLIN2 sense 5′- GCTGGGGATTACAGACCAGA-3′, PLIN2 antisense 5′- TCTTGGGGTTTTGGAAAATG-3′. Normalization was done to ChIP data with control IgG or was expressed as percentage input.

### Animal experiments

C57BL/6 mice (8 weeks of age) were purchased from Central Lab. Animal Inc. (Seoul, Korea) and housed and maintained on with a regular chow diet (ND: 10 kcal% fat, SLACOM, #M01). The mice were injected with a total of 1 × 10^9^ PFU recombinant adenovirus (Ad-GFP or Ad-JMJD2B) via tail vein injection. After injection, adenovirus-injected mice were fed a HFD for 2 weeks, whereas normal mice were fed a ND. Therefore, the mice were allocated to one of the three groups: mice without adenovirus injection + ND, mice injected with adenovirus containing GFP (Ad-GFP) + HFD (high-fat diet, 60% kcal from fat, TD.06414) (Harlan Teklad, IN, USA), and mice injected with adenovirus containing JMJD2B (Ad-JMJD2B) + HFD. Two weeks after injection, the mice were fasted for 6h, blood samples were harvested from the tail vein, and then animals were sacrificed for further analysis. All animal experiments were approved by Pusan National University Institutional Animal Care and Use Committee in accordance with the established ethical and scientific care procedures (PNU-2017-1483).

### Histological analysis and immunohistochemical staining

Mice liver tissues were dissected, fixed in 10% buffered formalin, and embedded in paraffin. A total of 10 sections (5 μm thick) were cut using a frozen microtome (HM560H, Microm Laboratory, Walldorf, Germany) and were stained with hematoxylin and eosin (H&E) and Oil Red O (ORO). For JMJD2B immunohistochemical staining, tissue sections were incubated in a solution of 0.3% H_2_O_2_ for 15 min. The sections were incubated with primary antibodies against JMJD2B (1:50 dilution) for 1 h at room temperature. Antibody binding was detected using the Envision system featuring anti-rabbit antibody (K4003, DAKO, Denmark). The slides were stained with liquid diaminobenzidine tetrahydrochloride (DAB+), a high-sensitivity substrate- chromogen system (K3468, DAKO, Glostrup, Denmark). Nuclei were counterstaining with Meyer’s hematoxylin. Images were acquired using an Olympus BX40 light microscope.

### Hepatic triglyceride (TG) measurement

TG was extracted from HepG2 cell suspensions (plated in 6-well plates at 4 ˆ 10^5^ cells per well) or liver tissue (100 mg) lysates in chloroform/methanol/H2O (8:4:3, v/v/v). The bottom layer (organic phase) obtained after centrifugation at 800 × g for 10 min and dried overnight. TG content were determined using commercial kits using an AM 157S-K TG kit (Asan Pharmaceutical, Seoul, Republic of Korea).

### Biochemical analysis from blood samples

Mice were sacrificed and blood samples were collected after fasting for 12 h. The collected samples were centrifuged at 1,000 × *g* for 15 min at 4 °C. The collected serum was stored at −80 °C until further analysis. The concentrations of total triglyceride and total cholesterol were measured by using a FUJI DRI-CHEM 700i (FUJI FILM, Japan).

### Statistical analysis

Data represent mean ± SEM of at least three independent experiments. The statistical significance between control and test groups was analyzed by using a two-tailed Student’s *t*-test. p-values < 0.05 were considered significant.

## Electronic supplementary material

Supplementary figures and table
