# Supplementary material for: Histone H3K9 demethylase JMJD2B induces hepatic steatosis through upregulation of PPARγ2
Source: Sci Rep. 2018 Sep 13;8:13734. doi: 10.1038/s41598-018-31953-x (PMC6137221; doi:10.1038/s41598-018-31953-x)
Supplement: Supplementary file 1 — Supplementary figures and table [file 41598_2018_31953_MOESM1_ESM.pdf]

Histone H3K9 demethylase JMJD2B induces hepatic steatosis through upregulation of PPARγ2

Ji-Hyun Kim[1], Dae Young Jung[1], Arulkumar Nagappan[1], Myeong Ho Jung[1*]

[1]School of Korean Medicine, Pusan National University, 49 Busandaehak-ro, Mulgeum-eup, Yangsan-si, Gyeongnam, 50612, Republic of Korea.

* Corresponding author: Myeong Ho Jung, School of Korean Medicine, Pusan National University, 49 Busandaehak-ro, Mulguem-eup, Yangsan-si, Gyeongnam, 50612, Republic of Korea

Tel.: 82-51-510-8468, Fax: 82-51-510-8437, E-mail: jung0603@pusan.ac.kr

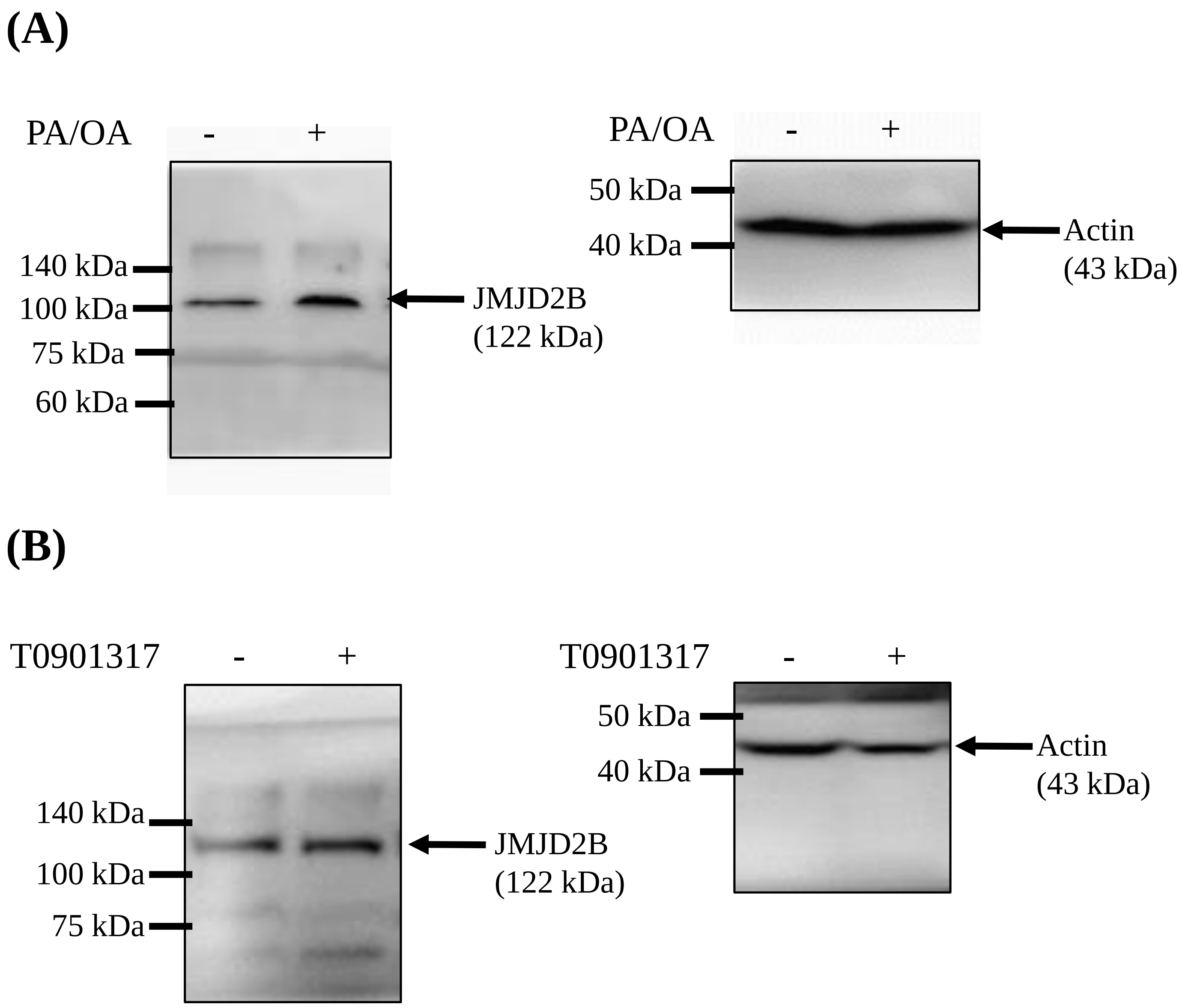

Supplementary Figure S1. Western blot of JMJD2B expression in PA/OA-treated HepG2 cells (A) and T0901317-treated HepG2 cells (B). The full length western blots of JMJD2B and actin from cell lysates are shown in the box panels. The western blots were derived from the same experimental conditions. Arrows indicate the JMJD2B and actin in Fig 1A and 1B.

(A)

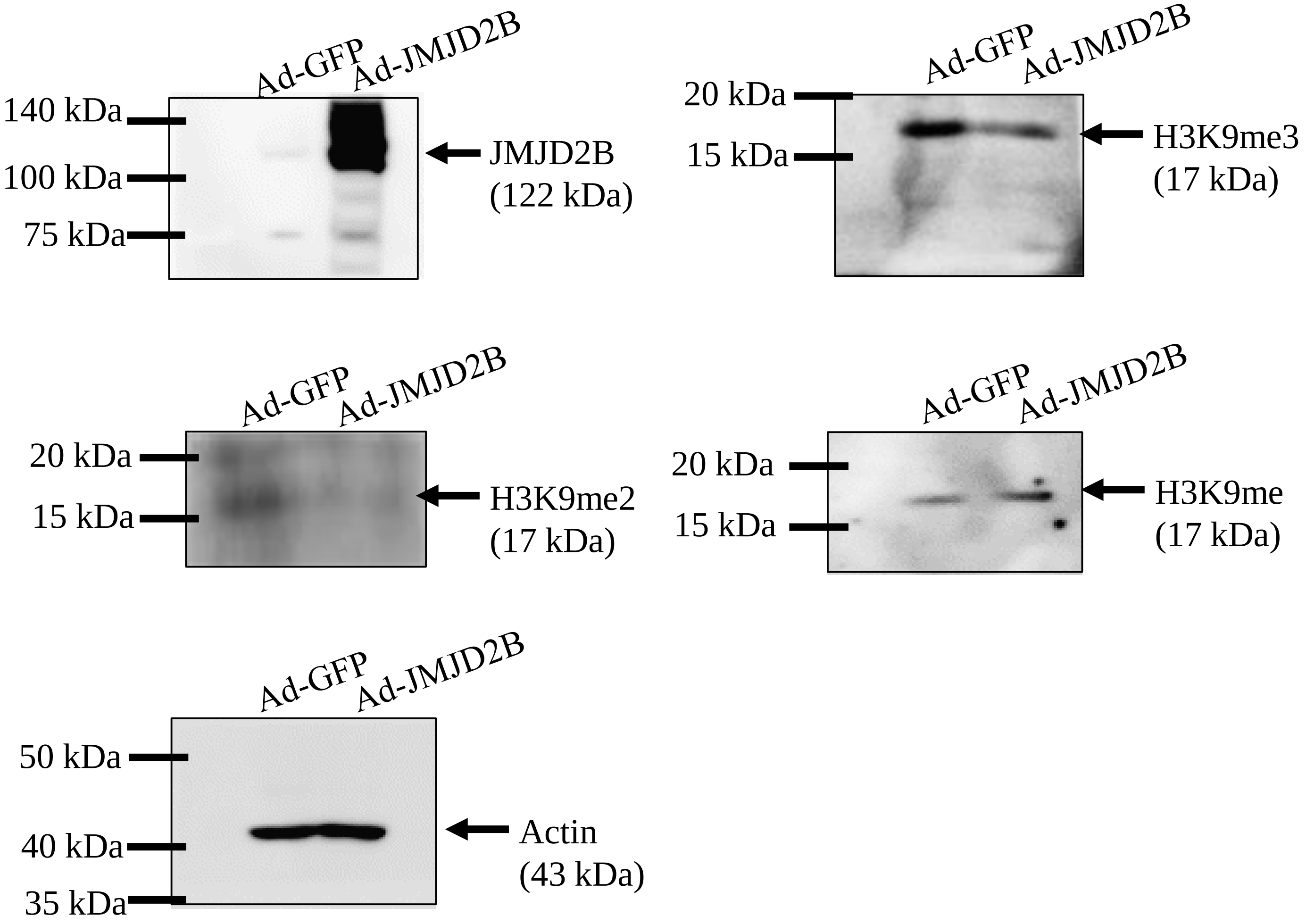

Supplementary Figure S2A. Western blot of JMJD2B, H3K9me3, H3K9me2, H3K9me and actin in Ad-JMJD2B-infected HepG2 cells. The full length western blots of JMJD2B, H3K9me3, H3K9me2, H3K9me and actin from cell lysates are shown in the box panels. The western blots were derived from the same experimental conditions. Arrows indicate JMJD2B, H3K9me3, H3K9me2, H3K9me and actin in Fig. 2A.

(B)

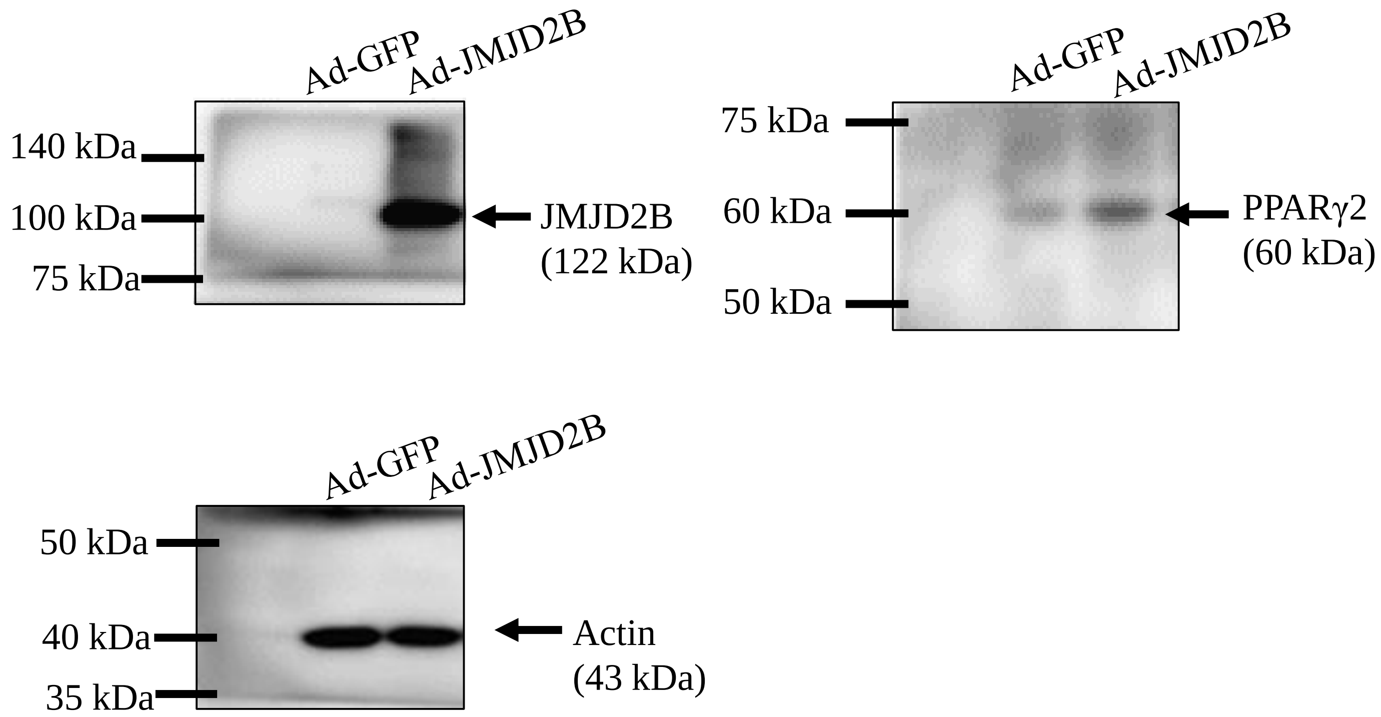

Supplementary Figure S2B. Western blot of JMJD2B, PPARγ2 and actin in Ad-JMJD2B-infected HepG2 cells. The full length western blots of JMJD2B, PPARγ2 and actin from cell lysates are shown in the box panels. The western blots were derived from the same experimental conditions. Arrows indicate JMJD2B, PPARγ2 and actin in Fig 2C.

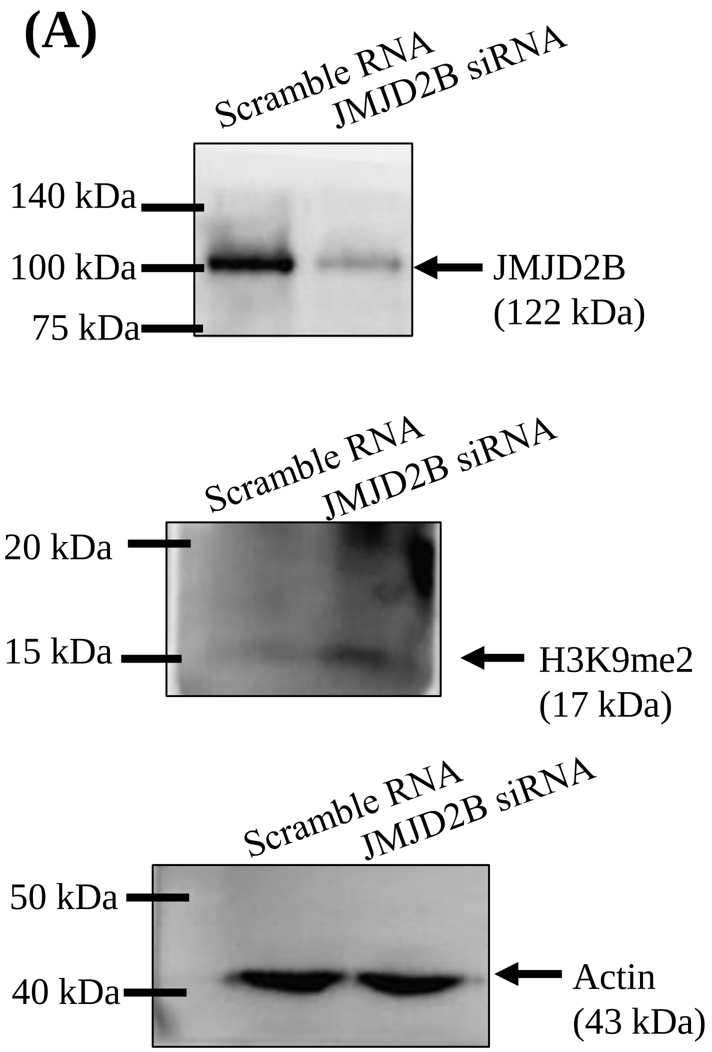

Supplementary Figure S3A. Western blot of JMJD2B, H3K9me3, H3K9me2, H3K9me and actin in JMJD2B siRNA-transfected HepG2 cells. The full length western blots of JMJD2B, H3K9me3, H3K9me2, H3K9me and actin from cell lysates are shown in the box panels. The western blots were derived from the same experimental conditions. Arrows indicate JMJD2B, H3K9me3, H3K9me2, H3K9me and actin in Fig. 3A.

(B)

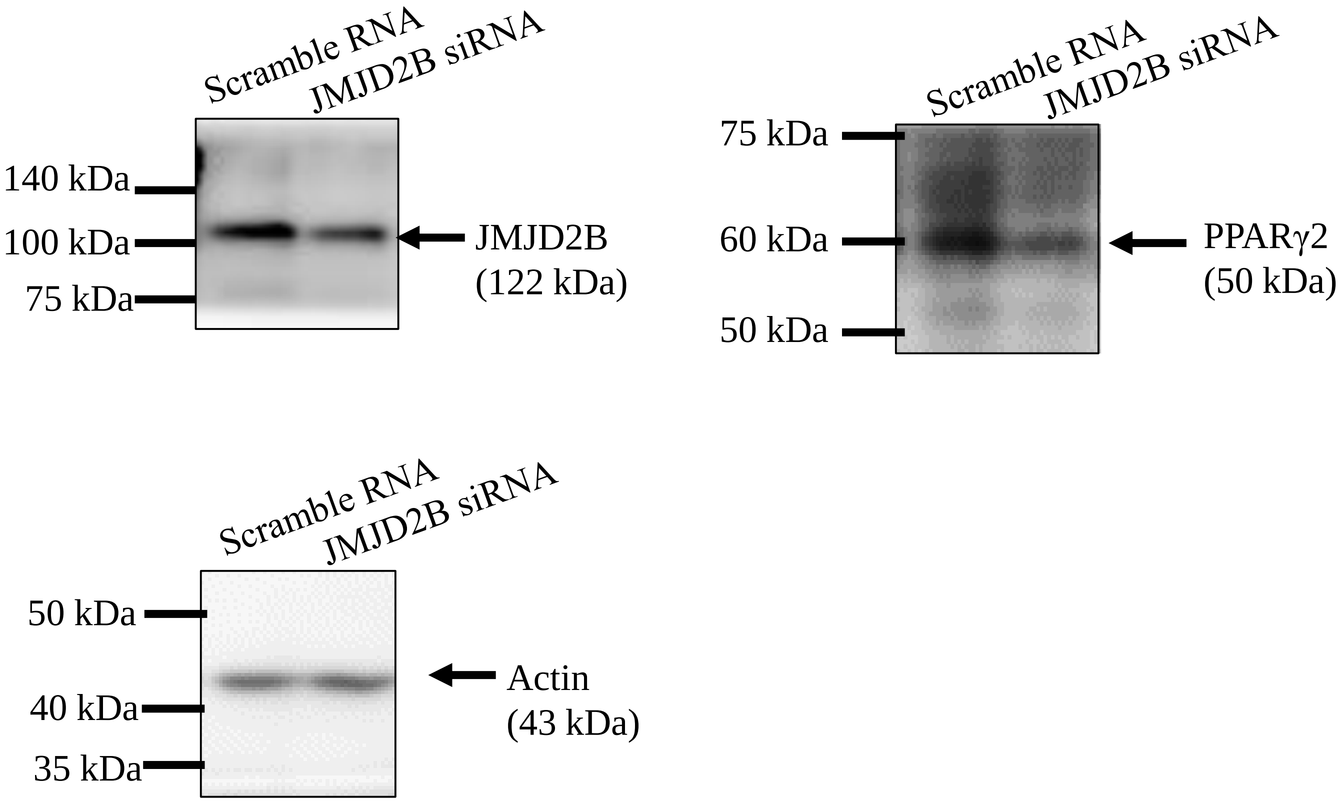

Supplementary Figure S3B. Western blot of JMJD2B, PPARγ2 and actin in JMJD2B siRNA-transfected HepG2 cells. The full length western blots of JMJD2B, PPARγ2 and actin from cell lysates are shown in the box panels. The western blots were derived from the same experimental conditions. Arrows indicate the JMJD2B, PPARγ2 and actin in Fig 3C.

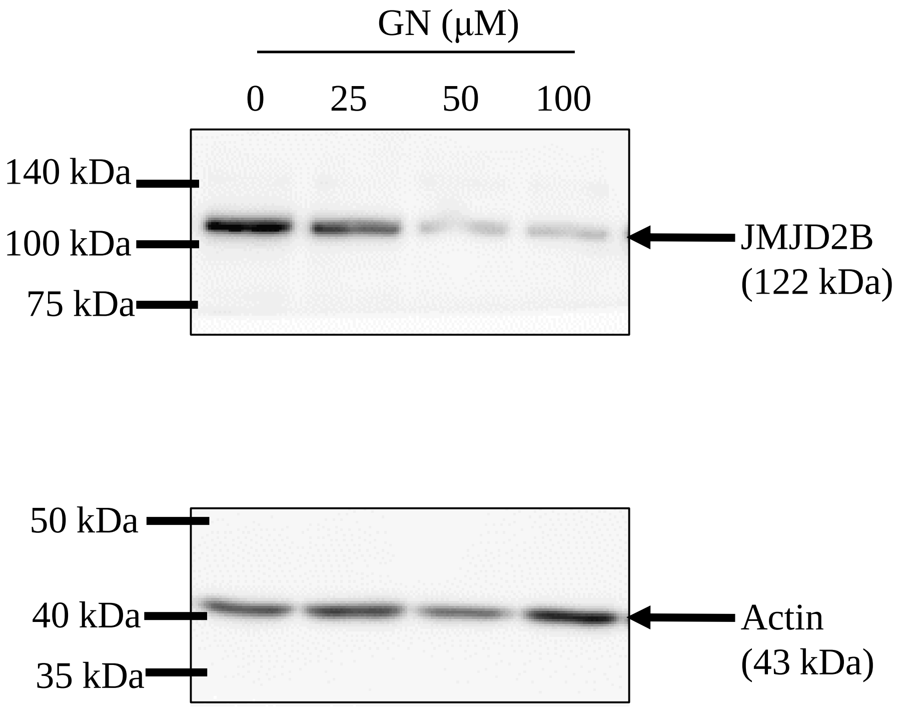

Supplementary Figure S4. Western blot of JMJD2B and actin in GN-treated HepG2 cells. The full length western blots of JMJD2B and actin from cell lysates are shown in the box panels. The western blots were derived from the same experimental conditions. Arrows indicate the JMJD2B and actin in Fig 7.

Supplementary Table S1. List of primers for q-PCR

Gene	Forward primer (5' → 3')	Reverse primer (5' → 3')
hJMJD2B	GGCCGGAGCTGCACACT	CGCGTCTTTGCACAGAGTAAGA
hPPARγ2	GGTGAA ACTCTGGGAGATTCT	CTCTGTGTCAACCATGGTCA
hCD36	CTTTGGCTTAATGAGACTGGGAC	GCAACAAACATCACCACACCA
hFABP4	TACTGGGCCAGGAATTTGAC	GTGGAAGTGACGCCTTTCAT
hPLIN2	CTCTCGATACACCGTGCAGA	TGGTCCTCATGATCCTCCTC
hCIDEC	TTGATGTGGCCCGTGTAACG	AAGCTTCCTTCATGATGCGC
h18S	CGGCTACCACATCCAAGGAA	GCTGGAATTACCGCGGCT
mJMJD2B	GGCCAAGATCATTCCACCCA	CCCACAGTCATGGCCTTCTT
mPPARγ2	GTGCCAGTTTCGATCCGTAGA	GGCCAGCATCGTGTAGATGA
mCD36	GAGCAACTGGTGGATGGTTT	GCAGAATCAAGGGAGAGCAC
mFABP4	GATGAAATCACCGCAGACGACA	ATTGTGGTCGACTTTCCATCCC
mPLIN2	GGCGTCTCTTTTCTCCAGGA	CGGATCCACTACTGCTGCTG
mCIDEC	AGGCCCTGTCGTGTTAGCAC	CATGATGCCTTTGCGAACCT
m18S	CGGCTACCACATCCAAGGAA	CGGCTACCACATCCAAGGAA
